# Connexin 43 is overexpressed in human fetal membrane defects after fetoscopic surgery†

**DOI:** 10.1002/pd.4917

**Published:** 2016-09-25

**Authors:** David W. Barrett, Anna L. David, Christopher Thrasivoulou, Alvaro Mata, David L. Becker, Alex C. Engels, Jan A. Deprest, Tina T. Chowdhury

**Affiliations:** ^1^ Institute of Bioengineering, School of Engineering and Materials ScienceQueen Mary University of LondonLondonUK; ^2^ Institute for Women's HealthUniversity College LondonLondonUK; ^3^ Department of Cell and Developmental BiologyUniversity College LondonLondonUK; ^4^ Lee Kong Chian School of MedicineNanyang Technological UniversitySingapore; ^5^ Department of Obstetrics and GynaecologyUniversity Hospitals LeuvenLeuvenBelgium

## Abstract

**Objective:**

We examined whether surgically induced membrane defects elevate connexin 43 (Cx43) expression in the wound edge of the amniotic membrane (AM) and drives structural changes in collagen that affects healing after fetoscopic surgery.

**Method:**

Cell morphology and collagen microstructure was investigated by scanning electron microscopy and second harmonic generation in fetal membranes taken from women who underwent fetal surgery. Immunofluoresence and real‐time quantitative polymerase chain reaction was used to examine Cx43 expression in control and wound edge AM.

**Results:**

Scanning electron microscopy showed dense, helical patterns of collagen fibrils in the wound edge of the fetal membrane. This arrangement changed in the fibroblast layer with evidence of collagen fibrils that were highly polarised along the wound edge but not in control membranes. Cx43 was increased by 112.9% in wound edge AM compared with controls (*p* < 0.001), with preferential distribution in the fibroblast layer compared with the epithelial layer (*p* < 0.01). In wound edge AM, mesenchymal cells had a flattened morphology, and there was evidence of poor epithelial migration across the defect. Cx43 and COX‐2 expression was significantly increased in wound edge AM compared with controls (*p* < 0.001).

**Conclusion:**

Overexpression of Cx43 in the AM after fetal surgery induces morphological and structural changes in the collagenous matrix that may interfere with normal healing mechanisms. © 2016 The Authors. *Prenatal Diagnosis* published by John Wiley & Sons, Ltd.

## Abstract 2

**What's Already Known About This Topic?**

- After fetoscopic surgery, the fetal membrane fails to heal spontaneously and the membrane defect is prone to iatrogenic PPROM.

**What Does ThisStudy Add?**

- We observed abnormal connexin 43 expression in the wound edge with preferential distribution in the mesenchymal cells compared with the epithelial cells.
- The overexpression of Cx43 may interfere with normal healing of the amniotic membrane after fetoscopic surgery.

## Introduction

Mechanical rupture of the fetal membranes can occur as a result of trauma and after invasive prenatal interventions such as open fetal surgery, fetoscopy or amniocentesis leading to iatrogenic preterm premature rupture of the fetal membrane (PPROM). The demand for fetal surgery is increasing as it has become evident that in some conditions, treatment *in utero* improves long‐term outcome. Fetoscopic laser ablation is now routinely performed for advanced twin‐to‐twin transfusion syndrome (TTTS), and fetal repair of congenital myelomeningocele has been shown to improve motor function postnatally.^1^, ^2^ However, spontaneous healing of the defect in the amniotic membrane (AM) does not occur after fetoscopic surgery, and a visible membrane defect is left, which is prone to rupture.^3^, ^4^, ^5^ PPROM complicates over 30% of fetal surgeries that are being used to treat abnormalities in the unborn baby. However, PPROM and subsequent preterm birth compromises the outcome of treated babies, reducing the clinical effectiveness of fetal surgery. There are no clinical solutions to improve healing of the fetal membranes after they rupture.^6^, ^7^, ^8^

Histologically, the fetal membranes consist of several distinctive layers and cell types within a 3D extracellular matrix network. The epithelial layer is the outermost layer of the fetal membrane and is composed of amniotic epithelial cells that secrete collagen types III and IV that form the basement membrane. The fibroblast layer is the thickest layer and consists of mesenchymal cells that secrete types I and III collagen to form the compact and spongy layers of the AM. Although the chorion is thicker than the AM, this tissue has greater tensile strength. However, it has been reported that repeated stretching of the amniochorion reduces the viscoelastic nature of the tissue, making it more susceptible to rupture.^9^, ^10^ The loss in mechanical resistance has been attributed to alterations in the collagen network within the compact, fibroblast and spongy layers of the AM which is why this tissue ruptures first.^9^, ^11^, ^12^, ^13^ The pathological mechanisms that cause collagen disruption in the AM involve multiple pathways that increase production of cytokines, matrix metalloproteinases (MMPs) and/or prostaglandins.^14^, ^15^, ^16^, ^17^ In animal models, tensile stretch increased myometrial expression of inflammatory factors involving cyclo‐oxygenase‐2 (COX‐2), the oxytocin receptor and the gap junction protein, connexin‐43 (Cx43).^18^, ^19^, ^20^ In human amniotic epithelial cells, application of 11% static stretch, induced activation of NF‐kB and AP‐1 leading to expression of COX‐2, MMPs and prostaglandin E_2_ (PGE_2_) production.^21^ Similarly, repetitive strain in human AM induced an inflammatory response, leading to tissue softening caused by alterations in proteoglycan, collagen and elastin content.^22^, ^23^, ^24^, ^25^, ^26^, ^27^, ^28^ We observed that cyclic tensile strain of human AM increased expression of Cx43.^28^ This important contractile responsive protein is a candidate upstream regulator of PGE_2_ that affects cell migration, proliferation and matrix organisation. Overexpression of Cx43 plays a central role in preventing the wound healing response in human diabetic wounds and venous leg ulcers.^29^, ^30^ We hypothesise that Cx43 could regulate cell function and matrix composition in the AM after iatrogenic trauma and promote remodelling mechanisms that interfere with the repair process and compromise fetal membrane integrity. The present study examined whether surgically induced membrane defects after fetoscopic surgery increase Cx43 expression in the wound edge of the AM and drives structural changes in collagen architecture.

## Methods

### Patient recruitment and sample collection

Human placentas (*n* = 12) were collected after caesarean section after written informed consent from women who had undergone fetal surgery at University College Hospital London and the University Hospitals of Leuven, Belgium. Ethical approval was granted by the Joint UCL/UCLH Committees on the Ethics of Human Research (Ref: 08/H0714/87) and the Ethics Committee at the KU Leuven (Ref: P008‐2011). Women underwent fetoscopic surgery for treatment of TTTS (*n* = 10), fetal congenital diaphragmatic hernia (CDH, *n* = 1) and twin reversed arterial perfusion sequence (TRAPS, *n* = 1). Fetal surgical procedures were performed between 15 + 0 and 28 + 1 weeks of gestation and between 1 to 145 days before birth (Table 1). During the surgery, the fetoscopic entry site was created by using a 10‐Fr Teflon cannula (Cook Medical, Strombeek Bever, Belgium) and pyramidal trocar device (Karl Storz, Tuttlingen, Germany). At delivery, the fetoscopic defect site was identified by careful macroscopic survey of the fetal membrane, and the tissue around the wound was carefully excised. For the open fetal surgery case, the hysterotomy wound was excised as part of the routine entry into the uterus at caesarean section, and then the membranes were stripped from the overlying myometrium. A control region in the fetal membrane that was aligned in the same axis as the fetal defect and was at least 5 cm away from the wound edge was excised along with 2 cm margins of full thickness membrane. Excess maternal blood was removed by washing the fetal membrane specimens (5×5 cm) with Earle's Balanced Salt Solution for 2 min (Sigma‐Aldrich, UK). Control and wound edge fetal membrane specimens were immediately fixed in 4% paraformaldehyde (PFA) for 2 h and stored in PBS prior to analysis by scanning electron microscopy (SEM). In addition, the AM was separated from the chorionic membrane (CM) by gentle traction. The control and wound edge AM specimens were either fixed in 4% PFA for 2 h or stored in RNA Later (Qiagen, Manchester, UK) prior to imaging and gene expression analysis, respectively.

**Table 1:** Clinical information for patients undergoing laser ablation therapy

Patient number	Maternal age	Indication for surgery	GA intervention (weeks + days)	GA delivery (weeks + days)	Time from surgery to delivery (days)	Type of analysis
1	39	TTTS stage I	24 + 0	35 + 5	82	IMF and SHG
2	28	TTTS stage I	19 + 3	29 + 4	71	IMF and SHG
3	28	FETO for CDH	28 + 1	32 + 6	33	IMF and SHG
4	32	TTTS stage III	26 + 5	26 + 6	1	IMF and SHG
5	35	TTTS stage II,	24 + 3	25 + 3	7	IMF and SHG
6	20	TTTS stage II	18 + 5	30 + 1	100	IMF and SHG
7	34	TRAPS	15 + 0	35 + 0	140	RT‐qPCR
8	33	TTTS stage III	22 + 2	23 + 0	5	IMF and SHG
9	29	TTTS stage III	21 + 6	35 + 5	97	RT‐qPCR
10	36	TTTS stage V	19 + 3	37 + 4	145	RT‐qPCR
11	33	TTTS stage III	25 + 3	31 + 5	44	RT‐qPCR
12	29	TTTS stage IV	19 + 5	29 + 5	70	IMF and SHG

All pregnancies were delivered by caesarean section.

TTTS, twin‐to‐twin transfusion syndrome; FETO, fetoscopic tracheal occlusion; CDH: congenital diaphragmatic hernia; TRAPS, twin reversed arterial perfusion sequence; GA, gestational age; IMF, immunofluoresence; SHG, second harmonic generation; RT‐qPCR, real‐time quantitative polymerase chain reaction.

### Fetal membrane characterisation

Scanning electron microscopy was used to evaluate the morphological characteristics of the AM and CM after fetoscopic interventions in wound edge specimens. Following fixation, the fetal membranes were washed with Milli‐Q® water before being passed through a graded ethanol series and then critical point dried (K850, Quorum Technologies, UK). The tissue specimens were mounted on 10 mm SEM mounting blocks and sputter coated with 30 nm gold particles before SEM analysis of the AM and CM (FEI Inspect™‐F50, The Netherlands).

### Immunostaining

Immunostaining was performed with whole‐mount control and wound edge AM specimens that had been fixed in 4% PFA. The AM tissue specimens were washed twice in PBS for 5 min and with 0.1 M Lysine and 0.1% Triton X100 in PBS for 40 min. Specimens were blocked for 1 h using 1% goat serum with PBS + 0.1% Triton X100. Primary antibodies for Cx43 (diluted 1:4000; Sigma, 6219) were incubated at room temperature overnight, as described (Wang CM, 2007). The tissues were washed with PBS and incubated with goat Alexa488 anti‐rabbit secondary antibody for 2 h at room temperature (1:400, Life Technologies). Secondary antibody incubation in the absence of primary antibody was used as a negative control. Tissues were counterstained for 20 min with 1 µg/ml of the nuclear dye DAPI (1:1000).

### Second harmonic generation and confocal imaging

Control and wound edge AM specimens were imaged by two‐photon confocal imaging on Leica SP8 with Coherent Chameleon Ultra, Ti Sapphire mode‐locked IR laser, as previously described.^31^ Briefly, Cx43 and second harmonic generation (SHG) imaging was performed at 920 nm excitation, 25×0.95 NA water immersion objective. The Cx43 signal was collected with the non‐descanned external HyD detector through a FITC emission filter (500–550 nm barrier filter). The collagen SHG signal was collected via the transmission detector and 450–470 nm barrier filter. The DAPI signal was collected sequentially with 405 nm excitation to avoid bleed‐through of nuclear signal into the Cx43 signal and emission signal via the confocal pinhole to the de‐scanned HyD detector between 400490 nm. Approximately 150 micron volumes were acquired through the whole thickness of the samples at 1.2 µm *z*‐section intervals. Maximum brightness projections were performed of the whole z‐stack. Parameters for laser power, detector gain and offset were kept constant for each sample, so that direct comparisons of the 8‐bit digital images could be made per patient to permit quantification.

### Image quantification and quantitative analysis

To characterise the direction of collagen alignment, an orientation distribution analysis using the directionality ImageJ plugin was performed. SHG images were converted to binary and 2D orientation analysis calculated using the local gradient orientation method. Cx43 immunostaining levels were quantitatively evaluated per tissue area using a well‐established pixel‐counting method.^32^ The images were converted to binary images using identical threshold values, and objects exceeding 2 pixels were counted to identify Cx43 positive pixels per tissue area (µm^2^) or per cell nuclei. For quantification of Cx43 plaque density, the values for fluorescence intensity were converted to height using the Image J interactive 3D surface plot plugin, where each spike represents a Cx43 plaque.

### Real‐time quantitative PCR

Total RNA was extracted from control and wound edge AM specimens using Trizol reagent and purified using RNeasy Mini Kit (Qiagen, Manchester, UK). RNA was treated with DNA‐free DNase for 20 min (Ambion Applied Biosystems, Warrington, UK). A total of 200 ng RNA was reverse transcribed using Enhanced Avian RT First Strand cDNA synthesis kit with oligo(dT)_23_ primer (Sigma Genosys, Cambridge, UK). For real‐time RT‐qPCR, each reaction was run in duplicate on a 96‐well plate containing 5‐µl SYBR green mastermix, 2.5‐µl cDNA and 2.5‐µl primer pairs. The following specific primer sequences were used: Cx43 sense: 5′‐CTCGCCTATGTCTCCTCCTG‐3′, antisense: 5′‐TTGCTCACTTGCTTGCTTGT‐3′; COL1A1 sense: 5′‐CCCCGAGGCTCTGAAGGT‐3′, antisense: 5′‐CACCAGCAATACCAGGAGCA‐3′; COX‐2 sense: 5′‐GGACAGGATTCTATGGAG‐3′, antisense: 5′‐GGATGTCAACACATAACTC‐3′; GAPDH sense: 5′‐TCTCTGCTCCTCCTGTTC‐3′; and antisense: 5′‐CGCCCAATACGACCAAAT‐3′ (Sigma Genosys, Cambridge, UK). The Mx3000P quantitative PCR instrument was used for real‐time detection of PCR products (Stratagene, Amsterdam, the Netherlands). Thermocycling conditions were 95 °C for 2 min, followed by denaturation of 40 cycles at 95 °C for 15 s, annealing at 60 °C for 1 min, and extension at 72 °C for 1 min. PCR efficiencies for optimal primer pair concentrations were derived from standard curves (*n* = 3) by preparing a ten‐fold serial dilution of cDNA from a sample that represented the control. The real‐time PCR efficiencies (E) of amplification for Cx43 was defined according to the relation, E = 10^[‐1/slope]^. The *R* ^2^ value of the standard curve exceeded 0.99 and revealed efficiency values ranging from 1.98 to 2 (98–100%). Primer specificity was verified by examining the melting curve. Relative quantification of Cx43 was estimated by normalizing the target to the reference gene, GAPDH and to the calibrator sample (patient matched control AM) by a comparative C_t_ approach. For each sample, the ratio of target ∆Ct and reference ∆Ct was calculated, as previously described. 33, 34 Ratios were expressed on a logarithmic scale (arbitrary units).

### Statistical analysis

Two‐way Analysis of Variance (anova) and the *post hoc* Bonferroni‐corrected *t*‐test were used to examine data for Cx43 protein and gene expression in control and wound edge AM. The number of replicates per patient for the control and wound edge AM are indicated in the figure legend. In all cases, a level of 5% was considered statistically significant (*p* < 0.05).

## Results

### Morphology of the fetal membranes after fetoscopic surgery

**Figure 1:**
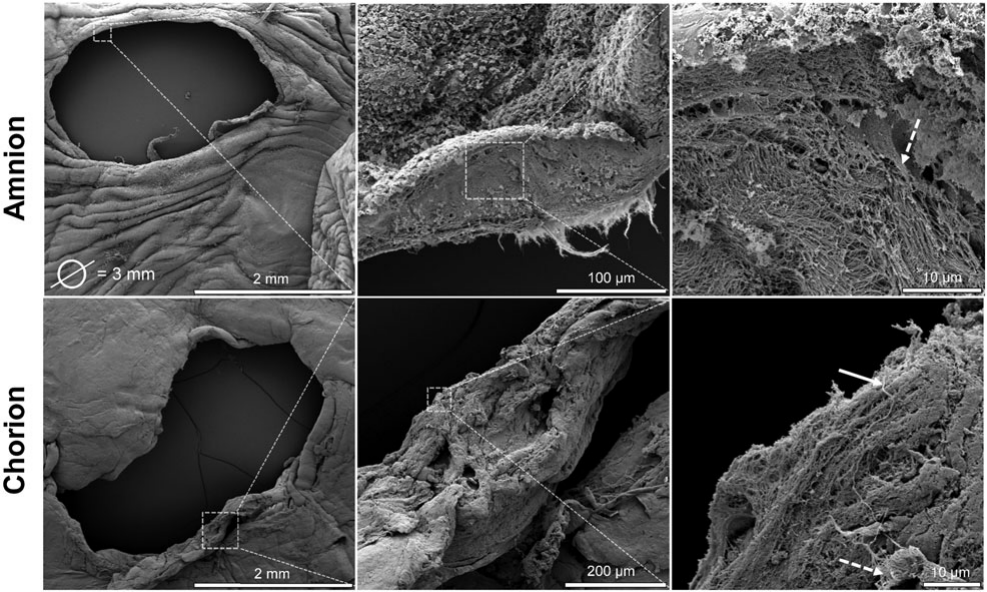
Scanning electron microscopy of fetal membrane defect. Image shows membrane surface and structural details within the amniotic membrane (top panel) and chorionic membrane (bottom panel) after fetoscopic surgery. The fetal membrane was taken from a 28‐year‐old patient who underwent fetoscopic surgery for treatment of stage I twin‐to‐twin transfusion syndrome at 19 weeks + 3 days and who was delivered at 29 weeks + 4 days by caesarean section. Scale bar indicated by white lines, with white boxes showing region of higher magnification

Figure 1 shows that there was no growth of tissue into the fetal membrane defect even 71 days after its creation. Instead, the wound edge was found to roll, resulting in an irregular wound size of approximately 3 mm diameter (Figure 1). The fetal defect site showed densely, packed, helical patterns of collagen fibrils in the AM and CM, with no preferential direction. In some cases, thinner and longer length fibres were observed in the CM (indicated by white arrow, Figure 1), but the microstructure mainly consisted of a discrete network of collagen along the wound edge. At higher magnification (right panel), the fibres appeared loose and randomly aligned in the matrix with some fibres that were intermingled (indicate by white dotted arrow; Figure 1).

### Collagen organisation in the fetal membrane defect

**Figure 2:**
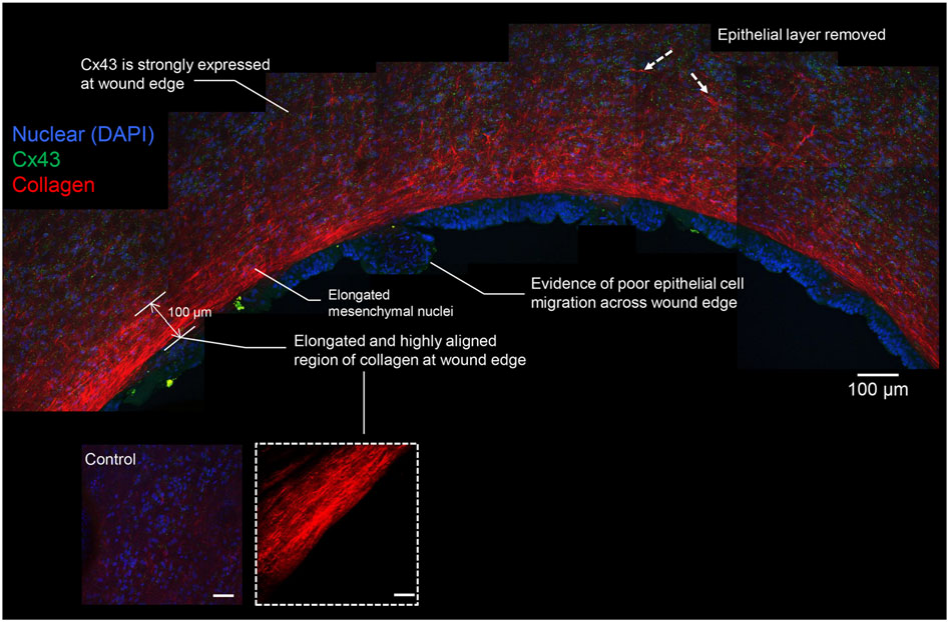
Collagen organisation and Cx43 protein expression in the amniotic membrane. The fetal membrane was taken from a 34‐year‐old patient who underwent fetoscopic laser coagulation in a monochorionic twin pregnancy complicated by twin reversed arterial perfusion. Gestational age at surgical intervention was 15 weeks + 0 days and gestational age at delivery by caesarean section was 35 weeks + 3 days. Merged images shows Cx43 expression in the fibroblast layer with dense regions of collagen fibres orientated along the length of the fibroblast layer in the wound edge of the amniotic membrane. Nuclei in blue were stained with DAPI and collagen fibres in red indicated by white lines detected by second harmonic generation confocal imaging. Inset shows higher magnification of the collagen fibres close to the wound edge. Merged image on left side shows control amniotic membrane taken from the same patient away from the wound. Scale bar = 100 µm

**Figure 3:**
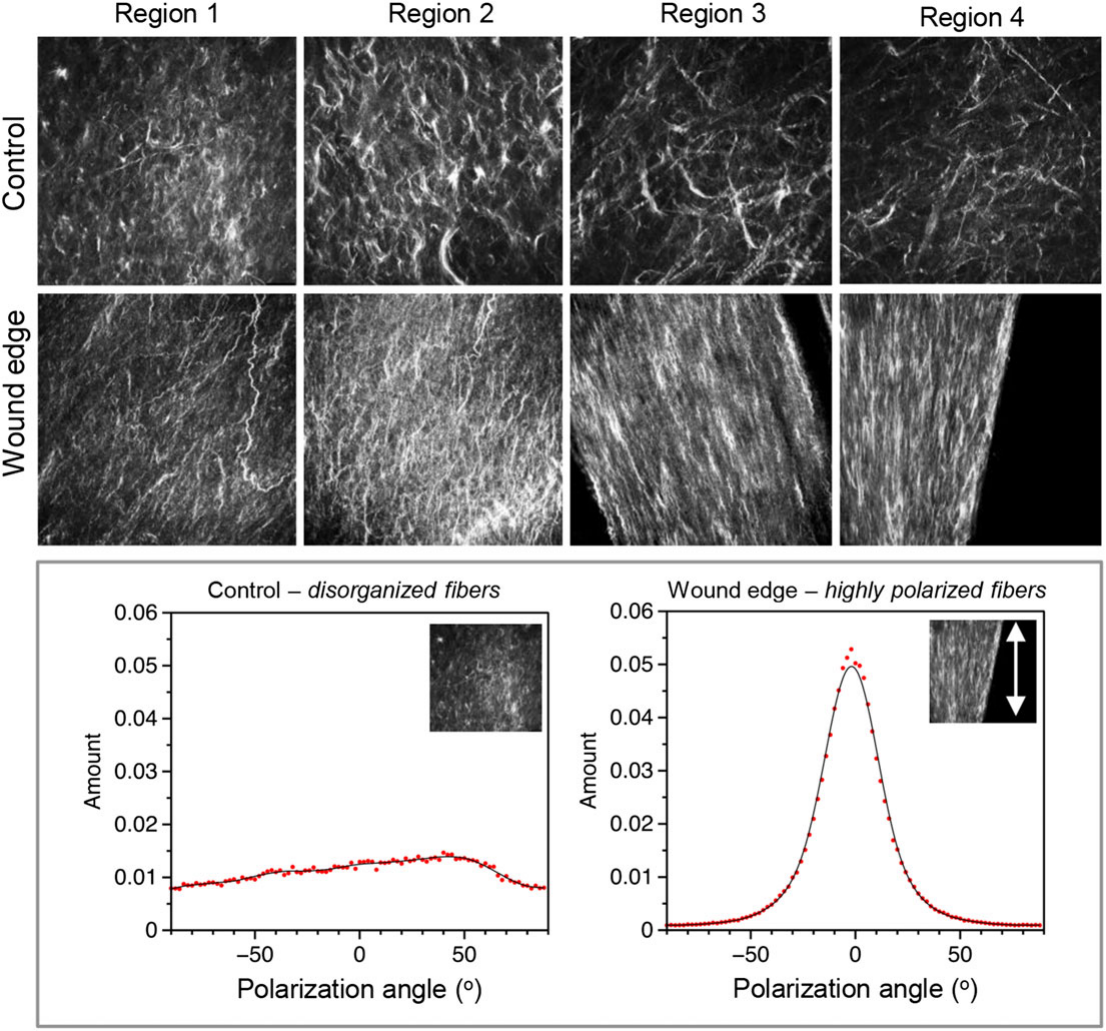
Collagen orientation in the amniotic membrane after fetoscopic surgery. Representative second harmonic generation confocal images show orientation of collagen fibres in four regions of the wound edge and compared to control amniotic membranes taken from the same patient away from the wound (top panel). A distribution of orientation values for each image is shown in the bottom panel with regions of highly polarised fibres in all regions of the wound edge compared with a disorganised arrangement in controls. The fetal membrane was taken from a 29‐year‐old patient who underwent fetoscopic surgery for treatment of stage IV twin‐to‐twin transfusion syndrome at 19 weeks + 5 days and who was delivered at 29 weeks + 5 days by caesarean section

Control and wound edge specimens were examined by SHG confocal microscopy to compare collagen structure in the matrix region of the AM following fetoscopic surgery. Figure 2 shows representative images of the AM wound edge region with evidence of collagen fibril bundle organisation in the basement membrane and compact layer (indicated by white dashed arrows). In contrast, this arrangement changes within the fibroblast and spongy layer with evidence of collagen fibrils that are much more organised and aligned in parallel to the axis of the wound edge. In the fibroblast and spongy layer, the formation of the collagen fibres appear dense, elongated and highly aligned (white dashed inset, Figure 2) with evidence of greater intensity of the SHG signal within a 100 µm region closest to the wound edge (indicated by white lines). The direction of collagen fibre organisation showed a region of highly polarised fibres in all regions of the wound edge with a spread of ~0° (Figure 3). This collagen structure appears to be more coherent and presents a different profile to control AM specimens where the fibre arrangement appears disorganised and is interwoven in a more or less random/loose fashion (Figure 3).

### Changes in cell morphology, Cx43 expression and collagen organisation

**Figure 4:**
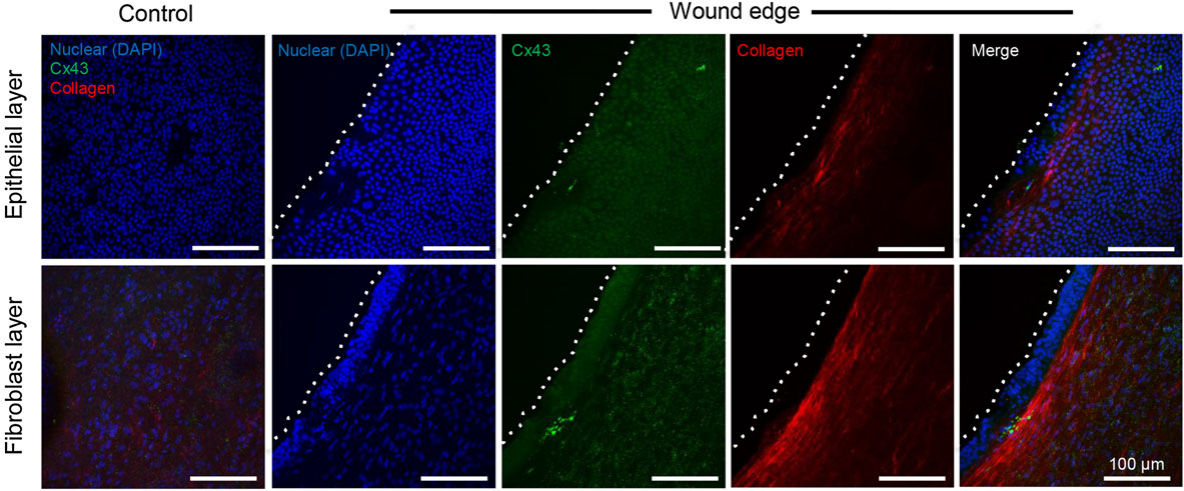
Collagen organisation and Cx43 protein expression in the epithelial and fibroblast layer. Merged images show characteristic spotted staining of Cx43 protein expression in the fibroblast layer of the amniotic membrane with dense regions of collagen fibres aligned in parallel along the wound edge of the amniotic membrane. Blue signal is DAPI staining of nuclei and collagen fibres in red indicated by white lines detected by second harmonic generation confocal imaging. The dotted white lines show the border along the length of the wound edge in the amniotic membrane. Merged image on left side shows control amniotic membrane taken from the same patient away from the wound. Scale bar = 100 µm

**Figure 5:**
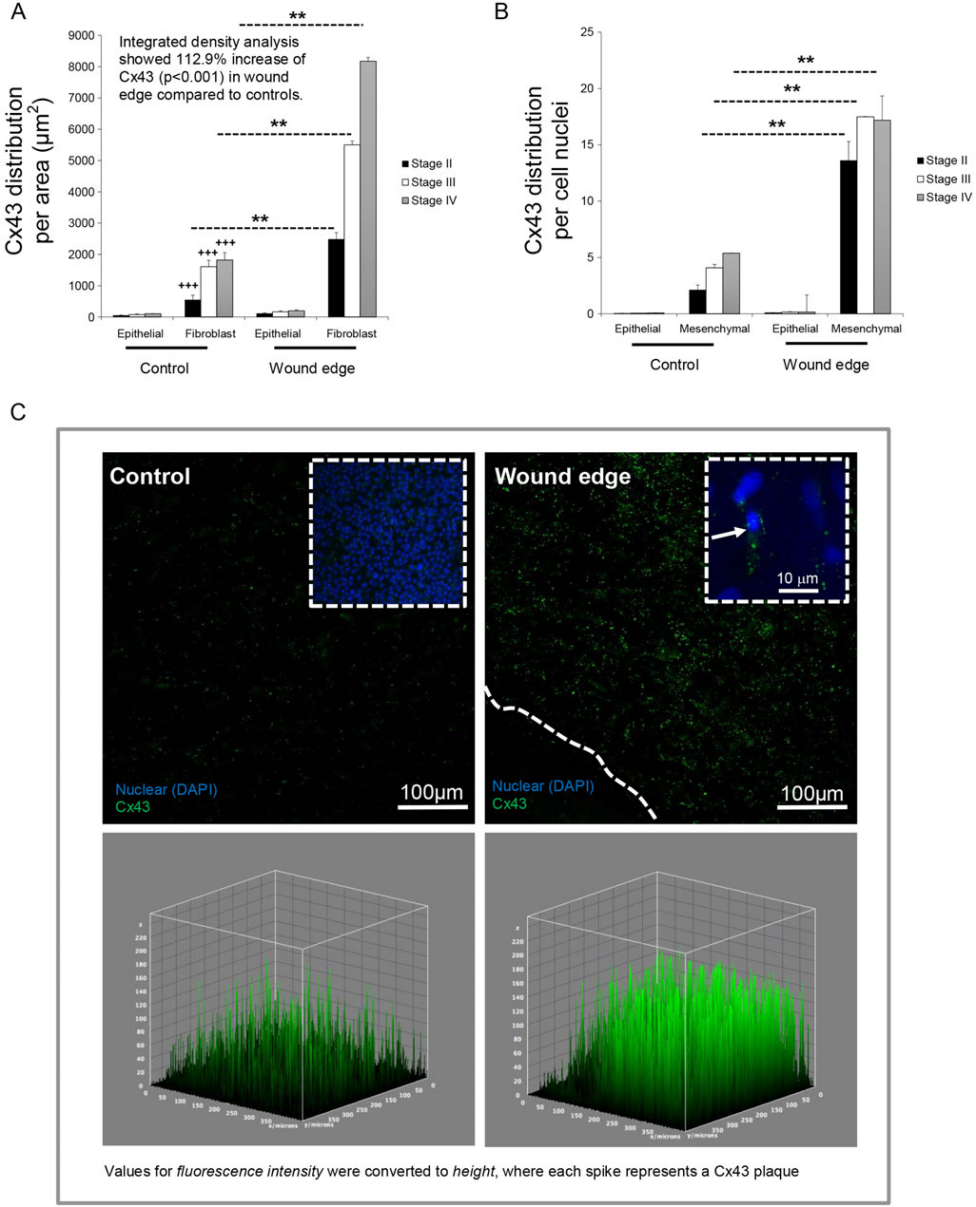
Cx43 distribution and plaque formation in the tissue layers of the amniotic membrane after fetoscopic surgery. The distribution of Cx43 was analysed per unit tissue area for comparisons between epithelial and fibroblast layer (A) and per cell nuclei (B). Integrated density was quantified for cell fluorescence and compared between control and wound edge specimens. For quantification of Cx43 plaque density, each spike represents a Cx43 plaque (C). In all cases, error bars represent the mean and SEM values for *n* = 8 to 10 replicates representing amniotic membranes taken from three patients, where *p < 0.05, **p < 0.01 and ***p < 0.001

**Figure 6:**
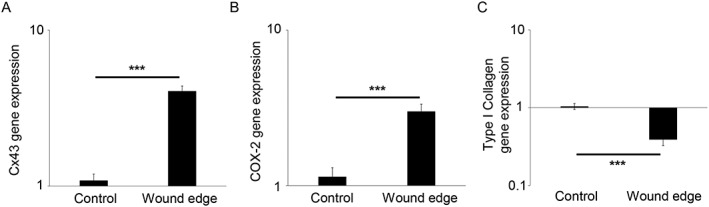
Gene expression levels in the amniotic membrane after fetoscopic surgery. Gene expression of Cx43, COX‐2 and Type I collagen was presented as ratio values and normalised to control amniotic membranes taken from the same patient away from the wound (B). In all cases, error bars represent the mean and SEM values for *n* = 12 to 18 replicates, representing amniotic membranes taken from three patients, where *p < 0.05, **p < 0.01 and ***p < 0.001

Our method of analysis by SHG in wound edge specimens used double imaging to examine qualitative changes in Cx43 protein expression by immunostaining of amniotic epithelial cells present in the epithelial layer and compared with mesenchymal cells present in the fibroblast layer, with nuclei counterstained with DAPI (Figure 4). In control AM, we visually observed low levels of Cx43 in regions within the epithelial and fibroblast layers (Figure 4). Quantitative analysis showed that the Cx43 signal was largely distributed in the fibroblast layer compared with the epithelial layer (all *p* < 0.001), with values ranging from 543.1 (stage 2) to 1818.6 µm^2^ (stage IV) for control AM (Figure 5A). In wound edge AM, a significantly greater proportion of total tissue area was occupied by Cx43 in the fibroblast layer with values that progressively increased with disease state (2479.5 to 8175.8 µm^2^; all *p* < 0.01). Furthermore, mesenchymal cells present in wounded AM showed a striking upregulation of Cx43 per cell nuclei compared with patient matched controls (all *p* < 0.01; Figure 5A), with negligible difference between diseased state. Integrated density analysis showed a significant increase of Cx43 protein expression in wound edge AM specimens (112.9%) compared with patient matched controls (*p* < 0.001). IMF confocal microscopy showed typical punctate plaques of Cx43 localised at the sites of cell–cell contacts with greater number of plaques distributed in the wounded fibroblast layer (Figure 5C) compared with control AM (inset). Gene expression of Cx43 and COX‐2 was significantly increased in wounded AM compared with patient matched controls and was paralleled with an associated reduction in Type I collagen (all *p* < 0.001; Figure 6). We did not observe significant differences in gene expression for control AM taken from individuals from the TTTS patient groups.

**Figure 7:**
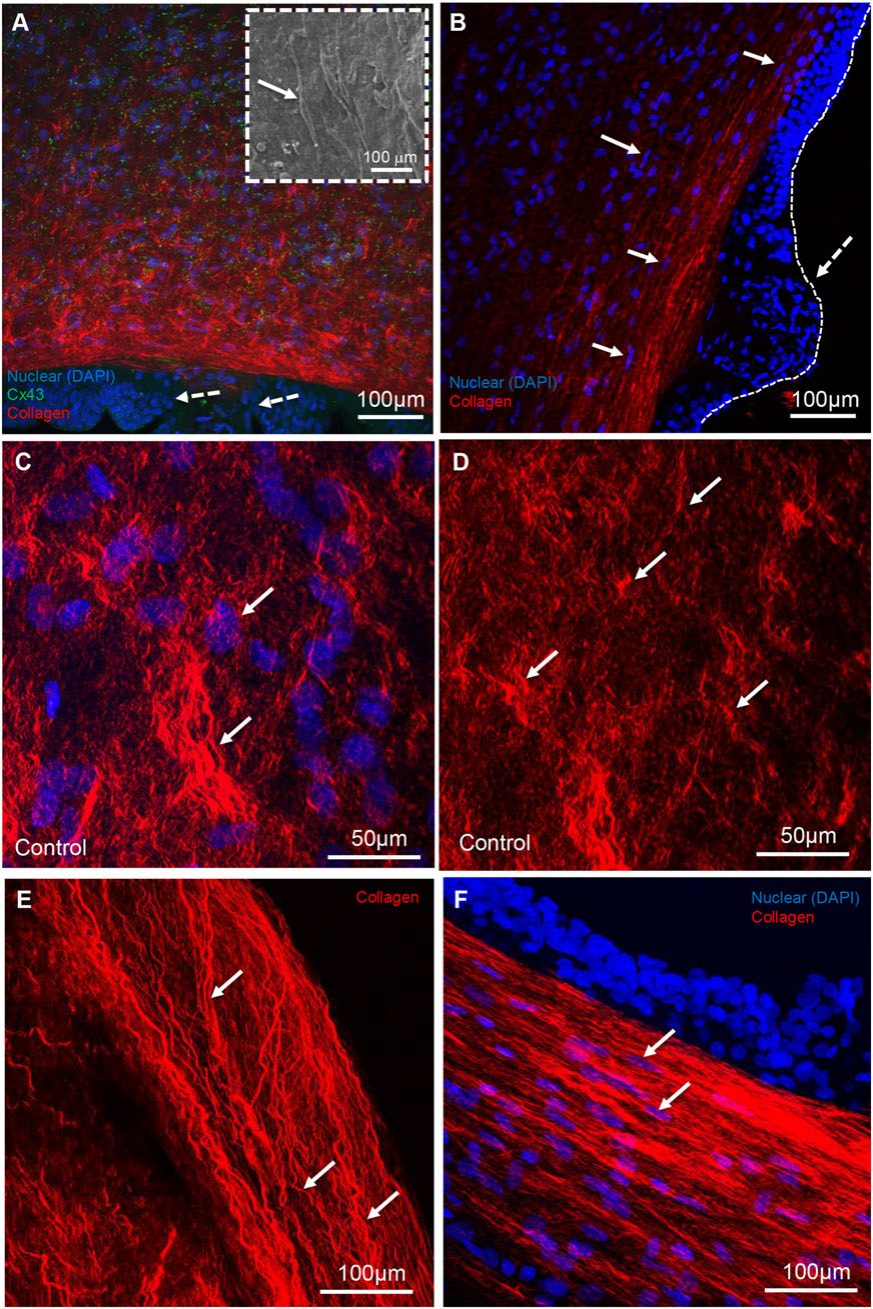
Cell migration and collagen organisation in the fetal membrane defect. Immunofluoresence confocal microscopy shows bulbous regions of epithelial cells (indicated by white dashed arrows, A, B) and increased Cx43 protein expression (D, E) and collagen alignment at the interface between the fibroblast layer and wound edge of the amniotic membrane (indicated by white arrows). Scanning electron microscopy shows flattened morphology of mesenchymal cells close to the wound edge with characteristic lamellipodium projections (C). Higher magnification shows typical punctate plaques of Cx43 at the cell to cell contacts (E) and densely aligned collagen fibres at the wound edge (F). Inset in D shows negligible Cx43 expression in fibroblast layer in patient matched control amniotic membrane. Wound edge in B and D is indicated by white dashed line. The thickness of the amniotic membrane is approximately 117 µm

To better understand the changes in cell behaviour and collagen organisation, we observed a thickened bulb region of amniotic epithelial cells with evidence of cell migration that formed a cell mass close to the wound edge (white dashed arrow, Figure 7A, B). Analysis by SEM showed a flattened mesenchymal cell morphology in the wounded fibroblast layer with characteristic lamellipodium projections (indicated by white arrows, Figure 7A inset) compared with a rounded‐like cell phenotype in control AM (Figure 7C). At higher magnification, we observed highly aligned collagen fibres dispersed with flattened mesenchymal cells along the wound edge (Figure 7E, F). In contrast, the arrangement of collagen in patient matched control AM was compact, irregular and basket‐like (Figure 7D).

## Discussion

Defects in the fetal membranes created by fetal surgery are a significant problem that limits the effectiveness of a variety of fetal surgical treatments. Several studies have shown that membrane damage caused by the insertion of a needle or fetoscope leads to inflammation and elevated levels of pro‐inflammatory cytokines, MMPs or C‐reactive protein.^35^ Histological studies have shown that wound repair is poor or absent in the fetal membranes, making the tissue less able to provide mechanical support.^36^, ^37^ This affects the signalling triggered by mechanotransduction, leading to major structural changes in the collagenous matrix as full term approaches.^13^ Thus, mechanisms to stimulate wound closure and repair of matrix tissues in the membranes are needed to withstand the effects of increased tension and prevent premature rupture. However, the pathological mechanisms that lead to diminished collagenous matrix synthesis and impair wound healing in fetal membranes are poorly understood. It is well established that overexpression of Cx43 reduced fibroblast migration and the healing response in diabetic skin and venous leg ulcers, but no study to date has explored whether this gap junction protein is expressed in the fetal AM and interferes with normal cell migration and collagen organisation across the defect.

The present study showed a striking upregulation of Cx43 in the wound edge of the AM defect after fetoscopic surgery, compared with patient matched control AM specimens taken 5 cm away from the site of trauma. This was accompanied by changes in cell morphology and augmented collagen deposition close to the wound edge, with distinct differences in Cx43 expression in the epithelial and fibroblast layer. Even with a small homogenous group of ten TTTS patients, we observed a significant upregulation of Cx43 that was dependent on disease state and may prevent efficient healing of the AM. However, our observations need to be studied in large groups of patients and with much more detail of the variables (e.g. maternal age, latency period and tissue location) that may influence membrane healing. Indeed, Papanna *et al.* used histology and immunohistochemistry techniques to compare the changes of the fetal membranes at the trocar insertion site with distance and observed differences in cell death, autophagy and structural integrity for fetal membranes taken from TTTS patients. The collagen architecture was severely altered in the referenced membranes and also in the recipient's fetal membranes. Because we did not examine these phenomena in *control* specimens taken at great distance away from the defect, it is difficult to determine the mechanism in the fetal membranes taken from different sites.^38^ Nevertheless, our observations in the human fetal AM are supported by evidence from animal models, which showed elevated expression of Cx43 in the wound edge of diabetic rats.^39^ In human diabetic foot ulcers, silencing Cx43 with antisense accelerated wound repair by improving fibroblast migration rates.^29^ Using *in vitro* organ culture models, it was reported that mimetic peptides that target Cx43 could improve fibroblast and keratinocyte migration rates leading to wound closure.^40^ Because it is possible to manipulate Cx43 expression, future studies should explore whether silencing of Cx43 in wounded membranes improves cell migration, with the aim to therapeutically control the self‐repair process.

We also observed poor epithelial migration adjacent to the site of injury across the defect. The mesenchymal cells appeared flattened in the fibroblast layer, and the epithelial cells crawled forward across the edge and formed a thickened bulb appearance, which may represent an initial attempt by the cells to heal the wound. Indeed, the formation of a thin tongue at the wound edge is an early hallmark of healing indicating the start of re‐epithelialisation. It is postulated that the efficiency of cell migration across the fetal defect could be influenced by the absence of factors that help the healing response. For example, cell proliferation and migration to the site of injury are regulated by integrins that activate physiological wound repair mechanisms. Although the regulation of integrins by tensile stretch in the AM is not known, apoptosis can result from the lack of integrin anchorage to the collagenous matrix.^41^, ^42^ Apoptosis and autophagy were observed in fetal membranes after fetoscopic surgery with histological changes that disrupt collagen structural integrity.^42^ The overexpression of Cx43 observed in the present study may play a role in switching off normal integrin signals and upregulate cytokines and enzymes that drive collagen degradation, leading to membrane rupture. In addition, stretching of the fetal membrane *in vitro* upregulates a number of pro‐inflammatory cytokines, chemokines and MMPs that trigger mechanotransduction and tissue remodelling, and these mechanisms should be explored further.

The present study showed greater deposition of collagen in the fibroblast and spongy layer compared with the epithelial regions, with highly polarised fibre alignment along the axis of the wound edge. Cell morphology appeared flattened in the wound edge, suggesting an attempt by mesenchymal cells to repair the tissue because of enhanced deposition of collagen along the axis of the fetal defect.^43^ This organization is reminiscent of embryonic wound healing where the wounds heal by contraction of an F‐actin band that forms around the wound and contracts in a purse string like fashion.^43^ In tenocytes subjected to tensile strain, there is evidence that Cx43 co‐localises with actin filaments and influences tissue remodelling and growth.^44^, ^45^ This reparative attempt of tissue remodelling may not contribute to normal collagen deposition needed for robust fetal membranes that can withstand tensile stretch during pregnancy. Indeed, previous *in vivo* models show that collagen structure can control the organisation of the connective tissue matrix. The remodelling phases of repair and arrangement of collagen fibres are dependent on the influence of mechanical forces that activate mechanotransduction pathways and modulate the quality of those fibres at the different levels of hierarchy.^20^ We previously found that subjecting the human AM to cyclic tensile strained increased collagen alignment and was mediated by overexpression of Cx43. This response was associated with a reduction in tangent stiffness and weakening of the AM. In the present study, the resulting alignment of fibroblasts and collagen could reduce elasticity in the membranes, which is less able to withstand the mechanical force along the wound edge. We did not determine the mechanical properties of the fetal membrane defect in this study because of limitations in the amount of tissue available for analysis. However, current mechanical approaches cannot easily separate the contribution of mechanical anisotropy because of the hierarchical arrangement of the collagen molecules in the fibril and/or parallel packing of fibrils in the tissue layers of the AM.^46^, ^47^, ^48^ The mechanical properties of collagen fibrils have been measured by nanoindentation and atomic force microscopy,^49^ suggesting that future studies, which examine fibril stiffening because of cell migration, should be investigated.

### Conclusion

The present study showed increased expression of Cx43 in the fibroblast and spongy layer of the human AM after fetoscopic surgery. We observed highly aligned collagen fibres along the axis of the wound edge secreted by mesenchymal cells that increased collagen deposition in an initial attempt to repair the AM. We postulate this effect is achieved by directing collagen matrix alignment and spatial arrangement similar to tissues, which have been subjected to tensile strain. However, the wound healing response is not sustainable long term and the molecular pathways activated by Cx43 to recruit matrix proteins and activate signalling mechanisms for repair should be investigated. We provide evidence that Cx43 plays a role in the process of wound healing in the human fetal membrane after fetoscopic surgery implicating regulation of this gap junction protein in directing collagen alignment.

## References

[1] Slaghekke F , Lopriore E , Lewi L , *et al.* Fetoscopic laser coagulation of the vascular equator versus selective coagulation for twin‐to‐twin transfusion syndrome: an open‐label randomised controlled trial. Lancet 2014;383(9935):2144–51. 24613024 10.1016/S0140-6736(13)62419-8

[2] Adzick NS , Thom EA , Spong CY , *et al.* A randomized trial of prenatal versus postnatal repair of myelomeningocoele. N Engl J Med 2011;364:993–1004. 21306277 10.1056/NEJMoa1014379 PMC3770179

[3] Deprest J , Van Schoubroeck D , Van Ballaer P , *et al.* Alternative access for fetoscopic Nd:YAG laser in TTS with anterior placenta. US Obstet Gynecol 1998;347–55. 10.1046/j.1469-0705.1998.11050347.x 9644775

[4] Gratacos E , Sanin‐Blair J , Lewi L , *et al.* A histological study of fetoscopic membrane defects to document membrane healing. Placenta 2006;27:452–6. 15953634 10.1016/j.placenta.2005.03.008

[5] Mallik AS , Fichter MA , Rieder S , *et al.* Fetoscopic closure of punctured fetal membranes with acellular human amnion plugs in a rabbit model. Obstet Gynecol 2007;110(5):1121–9. 17978128 10.1097/01.AOG.0000284624.23598.7c

[6] Deprest JA , Lerut TE , Vandenberghe K . Operative fetoscopy: new perspective in fetal therapy? Prenat Diagn 1997;17(13):1247–60. 9509543

[7] Harrison MR . Surgically correctable fetal disease. Am J Surg 2000;180(5):335–42. 11137683 10.1016/s0002-9610(00)00490-6

[8] Devlieger R , Millar LK , Bryant‐Greenwood G , *et al.* Fetal membrane healing after spontaneous and iatrogenic membrane rupture: a review of current evidence. Am J Obstet Gynecol 2006;195(6):1512–20. 16681986 10.1016/j.ajog.2006.01.074 PMC1665653

[9] Parry‐Jones E , Priya S . A study of the elasticity and tension of fetal membranes and the relation of the area of the gestational sac to the area of the uterine cavity. BJOG 1976;83:205–12. 10.1111/j.1471-0528.1976.tb00810.x 1252386

[10] Lavery JP , Miller CE , Knight RD . The effect of labor on the rheologic response of chorioamniotic membranes. Obstet Gynecol 1982;60(1):87–92. 7088455

[11] Jabareen M , Mallik AS , Bilic G , *et al.* Relation between mechanical properties and microstructure of human fetal membranes: an attempt towards a quantitative analysis. Eur J Obstet Gynecol Reprod Biol 2009;144:S134–41. 19282091 10.1016/j.ejogrb.2009.02.032

[12] Oyen M , Calvin SE , Cook RF . Uniaxial and biaxial mechanical behaviour of human amnion. Mater Res Soc Symp Proc 2005;844:161–6.

[13] Helmig R , Oxlund H , Petersen LK , Uldbjerg N . Different biomechanical properties of human fetal membranes obtained before and after delivery. Eur J Obstet Gynecol Reprod Biol 1993;48:183–9. 8335136 10.1016/0028-2243(93)90086-r

[14] Mercer BM . Preterm premature rupture of the membranes. Neuro Endocrinol Lett 2008;29(2):205–21.18404134

[15] Skinner KA , Challis JR . Changes in the synthesis and metabolism of prostaglandins by human fetal membranes and decidua at labour. Am J Obstet Gynaecol 1985;151:519–23. 10.1016/0002-9378(85)90281-9 3856386

[16] Devlieger R , Deprest JA , Gratacós E , *et al.* Matrix metalloproteinases ‐2 and ‐9 and their endogenous tissue inhibitors in fetal membrane repair following fetoscopy in a rabbit model. Mol Hum Reprod 2000;6(5):479–85. 10775654 10.1093/molehr/6.5.479

[17] Kumar D , Fung W , Moore RM , *et al.* Proinflammatory cytokines found in amniotic fluid induce collagen remodelling, apoptotis, and biophysical weakening of cultured human fetal membranes. Biol Reprod 2006;74:29–34. 16148217 10.1095/biolreprod.105.045328

[18] Parry LJ , Bathgate RA . The role of oxytocin and regulation of uterine oxytocin receptors in pregnant marsupials. Exp Physiol 2000;85:91S–99S. 10795911 10.1111/j.1469-445x.2000.tb00012.x

[19] Ou CW , Orsino A , Lye SJ . Expression of connexin‐43 and connexin‐26 in the rat myometrium during pregnancy and labor is differentially regulated by mechanical and hormonal signals. Endocrinology 1997;138:5398–5407. 9389525 10.1210/endo.138.12.5624

[20] Kendal‐Wright CE . Stretching, mechanotransduction, and proinflammatory cytokines in the fetal membranes. Reprod Sci 2007;14(8 Suppl):35–41. 18089608 10.1177/1933719107310763

[21] Mohan AR , Sooranna SR , Lindstrom TM , *et al.* The effect of mechanical stretch on COX‐2 expression and AP‐1 and NFĸB activity in human amnion cells. Endocrinology 2007;148:1850–7. 17218407 10.1210/en.2006-1289

[22] Sooranna SR , Engineer N , Loudon JAZ , *et al.* The mitogen‐activated protein kinase dependent expression of prostaglandin H synthase‐2 and interleukin‐8 messenger ribonucleic acid by myometrial cells: the differential effect of stretch and IL‐1β. J Clin Endocrinol Metab 2005;90:3517–27. 15784717 10.1210/jc.2004-1390

[23] Kanefsky J , Lenbury M , Hai CM . Cholingeric receptor and cyclic stretch‐mediated inflammatory gene expression in intact ASM. Am J Respir Cell Mol Biol 2006;34:417–25. 16339998 10.1165/rcmb.2005-0326OC PMC2644203

[24] Li LF , Ouyang B , Choukroun G , *et al.* Stretch‐induced IL‐8 depends on c‐Jun NH2‐terminal and nuclear factor‐kB inducing kinases. Am J Physiol Lung Cell Mol Physiol 2003;285(2):L464–75. 12716652 10.1152/ajplung.00031.2003

[25] Nemeth E , Millar LK , Bryant‐Greenwood GD . Fetal membrane distension: II. Differentially expressed genes regulated by acute distension *in vitro* . Am J Obstet Gynecol 2000;182:60–7. 10649157 10.1016/s0002-9378(00)70491-1

[26] McLaren J , Taylor DJ , Bell SC . Prostaglandin E(2)‐dependent production of latent matrix metalloproteinase‐9 in cultures of human fetal membranes. Mol Hum Reprod 2000;6(11):1033–40. 11044467 10.1093/molehr/6.11.1033

[27] Bryant‐Greenwood G . The extracellular matrix of the human fetal membranes: structure and function. Placenta 1998;19:1–11. 9481779 10.1016/s0143-4004(98)90092-3

[28] Chowdhury B , David AL , Thrasivoulou C , *et al.* Tensile strain increased COX‐2 expression and PGE_2_ release leading to weakening of the human amniotic membrane. Placenta 2014;35(12):1057–64. 25280972 10.1016/j.placenta.2014.09.006

[29] Mori R , Power KT , Wang CM , *et al.* Acute downregulation of Cx43 at wound sites leads to a reduced inflammatory response, enhanced keratinocyte proliferation and wound fibroblast migration. J Cell Sci 2006;119:5193–203. 17158921 10.1242/jcs.03320

[30] Mendoza‐Naranjo A , Cormie P , Serrano AE , *et al.* Overexpression of the gap junction protein Cx43 as found in diabetic foot ulcers can retard fibroblast migration. Cell Biol Int 2012;36(7):661–7. 22455314 10.1042/CBI20110628

[31] Theodossiou TA , Thrasivoulou C , Ekwobi C , Becker DL . Second harmonic generation confocal microscopy of collagen type I from rat tendon cryosections. Biophys J 2006;91(12):4665–77. 17130233 10.1529/biophysj.106.093740 PMC1779934

[32] Wang CM , Lincoln J , Cook JE , Becker DL . Abnormal connexin expression underlies delayed wound healing in diabetic skin. Diabetes 2007;56:2809–17. 17717278 10.2337/db07-0613

[33] Lee DA , Brand J , Salter DM , *et al.* Quantification of mRNA using real‐time PCR and Western blot analysis of MAPK events in chondrocyte/agarose constructs. Methods Mol Biol 2011;695:77–97. 21042967 10.1007/978-1-60761-984-0_6

[34] Pfaffl MW , Horgan GW , Dempfle L . Relative expression software tool (REST) for group wise comparison and statistical analysis of relative expression results in real time PCR. Nucleic Acids Res 2002;30:e3. 10.1093/nar/30.9.e36 PMC113859 11972351

[35] Gratacós E , Devlieger R , Decaluwé H , *et al.* Is the angle of needle insertion influencing the created defect in human fetal membranes? Evaluation of the agreement between specialists' opinions and *ex vivo* observations. Am J Obstet Gynecol 2000;182(3):646–9. 10739523 10.1067/mob.2000.103218

[36] Gratacós E , Sanin‐Blair J , Lewi L , *et al.* A histological study of fetoscopic membrane defects to document membrane healing. Placenta 2006;27(4–5):452–6. 15953634 10.1016/j.placenta.2005.03.008

[37] Millar LK , Stollberg J , DeBuque L , Bryant‐Greenwood G . Fetal membrane distention: determination of the intrauterine surface area and distention of the fetal membranes preterm and at term. Am J Obstet Gynecol 2000;182(1 Pt 1):128–34. 10649167 10.1016/s0002-9378(00)70501-1

[38] Papanna R , Mann LK , Moise KJ Jr , *et al.* Histologic changes of the fetal membranes after fetoscopic laser surgery for twin–twin transfusion syndrome. Pediatr Res 2015;78(3):247–55. 26020146 10.1038/pr.2015.105

[39] Qiu C , Coutinho P , Frank S , *et al.* Targeting connexin43 expression accelerates the rate of wound repair. Curr Biol 2003;30;13(19):1697–703. 10.1016/j.cub.2003.09.007 14521835

[40] Wright CS , van Steensel MA , Hodgins MB , Martin PE . Connexin mimetic peptides improve cell migration rates of human epidermal keratinocytes and dermal fibroblasts *in vitro* . Wound Repair Regen 2009;17(2):240–9. 19320893 10.1111/j.1524-475X.2009.00471.x

[41] Ruoslahti E , Engvall E . Integrins and vascular extracellular matrix assembly. J Clin Invest 1997;100(11 Suppl):S53–6. 9413402

[42] Divers MJ , Bulmer JN , Miller D , Lilford RJ . Beta 1 integrins in third trimester human placentae: no differential expression in pathological pregnancy. Placenta 1995;16:245–60. 7543673 10.1016/0143-4004(95)90112-4

[43] Redd MJ , Cooper L , Wood W , *et al.* Wound healing and inflammation: embryos reveal the way to perfect repair. Philos Trans R Soc Lond B Biol Sci 2004;359(1445):777–84. 15293805 10.1098/rstb.2004.1466 PMC1693361

[44] Wall ME , Otey C , Qi J , Banes AJ . Connexin 43 is localized with actin in tenocytes. Cell Motil Cytoskeleton 2007;64(2):121–30. 17183550 10.1002/cm.20170

[45] Stanley RL , Fleck RA , Becker DL , *et al.* Gap junction protein expression and cellularity: comparison of immature and adult equine digital tendons. J Anat 2007;211(3):325–34. 17848160 10.1111/j.1469-7580.2007.00781.x PMC2375813

[46] Graham JS , Vomund AN , Phillips CL , Grandbois M . Structural changes in human type I collagen fibrils investigated by force spectroscopy. Exp Cell Res 2004;299(2):335–42. 15350533 10.1016/j.yexcr.2004.05.022

[47] Eppell SJ , Smith BN , Kahn H , Ballarini R . Nano measurements with micro‐devices: mechanical properties of hydrated collagen fibrils. J R Soc Interface 2006;22;3(6):117–21. 10.1098/rsif.2005.0100 PMC1618494 16849223

[48] van der Rijt JA , van der Werf KO , Bennink ML , *et al.* Micromechanical testing of individual collagen fibrils. Macromol Biosci 2006;6(9):697–702. 16967482 10.1002/mabi.200600063

[49] Wenger MP , Bozec L , Horton MA , Mesquida P . Mechanical properties of collagen fibrils. Biophys J 2007;93(4):1255–63. 17526569 10.1529/biophysj.106.103192 PMC1929027

